# Investigation of the “Antigen Hook Effect” in Lateral Flow Sandwich Immunoassay: The Case of Lumpy Skin Disease Virus Detection

**DOI:** 10.3390/bios12090739

**Published:** 2022-09-08

**Authors:** Simone Cavalera, Giulia Pezzoni, Santina Grazioli, Emiliana Brocchi, Stefano Baselli, Davide Lelli, Barbara Colitti, Thea Serra, Fabio Di Nardo, Matteo Chiarello, Valentina Testa, Sergio Rosati, Claudio Baggiani, Laura Anfossi

**Affiliations:** 1 Department of Chemistry, University of Turin, Via Pietro Giuria 7, 10137 Turin, TO, Italy; 2 Istituto Zooprofilattico Sperimentale della Lombardia e dell’Emilia-Romagna, National/OIE/FAO Reference Centre for FMD and SVD, Via Antonio Bianchi 7, 25124 Brescia, BS, Italy; 3 Department of Veterinary Science, University of Turin, Largo Paolo Braccini 2, 10095 Grugliasco, TO, Italy

**Keywords:** point-of-care test, rapid diagnosis, infectious diseases, single-epitope sandwich, double-epitope sandwich, lumpy skin disease

## Abstract

Lumpy skin disease (LSD) is an infectious disease affecting bovine with severe symptomatology. The implementation of effective control strategies to prevent infection outbreak requires rapid diagnostic tools. Two monoclonal antibodies (mAbs), targeting different epitopes of the LSDV structural protein p32, and gold nanoparticles (AuNPs) were used to set up a colorimetric sandwich-type lateral flow immunoassay (LFIA). Combinations including one or two mAbs, used either as the capture or detection reagent, were explored to investigate the hook effect due to antigen saturation by the detector antibody. The mAb-AuNP preparations were optimized by a full-factorial design of experiment to achieve maximum sensitivity. Opposite optimal conditions were selected when one Mab was used for capture and detection instead of two mAbs; thus, two rational routes for developing a highly sensitive LFIA according to Mab availability were outlined. The optimal LFIA for LSDV showed a low limit of detection (10^3.4^ TCID_50_/mL), high inter- and intra-assay repeatability (CV% < 5.3%), and specificity (no cross-reaction towards 12 other viruses was observed), thus proving to be a good candidate as a useful tool for the point-of-need diagnosis of LSD.

## 1. Introduction

**Figure 1:**
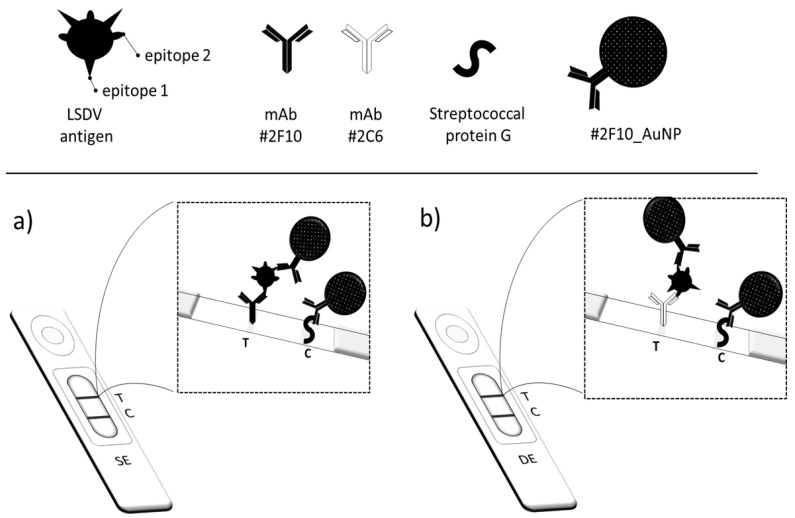
Schematic of the two sandwich-type immunoassays using: one antibody (single epitope, (**a**)) and two different antibodies (double epitope, (**b**)) as capture and detection.

In the recent years, more than ever, screening tests used for monitoring infectious diseases have been potentiated to counteract pathogenic local outbreaks, as well as pandemics [1,2,3]. The World Health Organization (WHO) has suggested increasing the use of the point-of-care tests (POCTs), as they optimally fit with criteria gathered under the acronym of (RE)ASSURED (Real-time connectivity, Easy of specimen collection, Affordability, Sensitivity, Specificity, User-friendliness, Rapidness/Robustness, Equipment-free, Deliverability) [4]. Among analytical strategies involved in POCTs, the most diffuse and successful technique is the immunochromatographic strip test, also known as the lateral flow immunoassay (LFIA). Many POCTs in the LFIA format are routinely employed to monitor infectious diseases by the Centers for Disease Control and Prevention (CDC) [5,6]. As well as any kind of immunoassay, the LFIA exploits the affinity of specific antibodies to detect antigens from viruses, fungi, or bacteria, thus enabling the direct diagnosis of the infection. The LFIA for antigen detection is based on the formation of the immunocomplex between a labeled specific antibody (detection antibody) and the target antigen, and its capture by another specific antibody (capture antibody) anchored to a support to create a reactive zone (test line). The signal is generated by the accumulation of the labeled antibody and antigen immunocomplex in correspondence to the test line. For colorimetric LFIAs, the label is a colored nanomaterial, such as metal (e.g., Au, Ag), latex, or carbon nanoparticles [7,8]. As a widespread and impactful infectious disease affecting bovines, lumpy skin disease (LSD) needs an effective control strategy to limit the spread of the infection. The etiological agent is the lumpy skin disease virus (LSDV), a large double-stranded DNA virus with an oval or brick-shaped morphology, and a genome of about 150 kDa. LSDVs belong to the *Capripoxvirus genus*, of the *Poxviridae* family. LSD causes economic damage, such as loss in milk production, besides limping and more severe symptomatology [9,10]. During the 2012–2018 Eurasian LSD epidemic, the morbidity and mortality associated with the epidemic were reported at 9% to 26% and 0.5% to 2%, respectively [11]. Though characteristic clinical signs of LSD enable a presumptive diagnosis, laboratory confirmation is necessary for the successful control and eradication of LSD, particularly in cases of mild disease. Laboratory confirmation of LSD relies on electron microscopy examination, virus isolation (VI), serological tests, and polymerase chain reaction (PCR). Viral isolation is the gold standard for LSDV diagnosis; however, it requires several weeks to isolate the virus. Molecular methods, including conventional or real-time polymerase chain reaction and loop-mediated isothermal amplification, are the most sensitive methods for detecting LSDV [12,13,14,15,16,17,18,19]. As far as immunodiagnostics is concerned, serological assays are available to indirectly diagnose LSDV by detecting the immune response to the virus in the blood of infected animals, while assays aimed at detecting viral antigens have not been reported yet, with the exception of the immunohistochemical method for detecting the LSDV antigens in skin nodules of infected cattle [20]. However, there is a lack of simpler virological tests, such as ELISA, for antigen detection or rapid tests enabling the point-of-need diagnosis of LSD, which would be particularly useful in endemic countries. In a previous study, monoclonal antibodies (mAbs) raised against the Neethling strain of LSDV and recognizing different epitopes of a 35 kDa viral protein (LSDV p32) were shown to detect LSDV isolates when used as coating and peroxidase-conjugated antibodies in a sandwich ELISA format [21]. Therefore, taking advantage of these well-characterized pairs of mAbs, here we aimed at developing a sensitive colorimetric LFIA for the on-field and visual detection of the LSD virus in clinical samples. Though apparently antibodies can be employed for capture or detection of target antigens, optimizing the role of each bioreagent modulated the performance of the LFIA device [22]. In addition, other parameters, such as the antibody-to-label ratio, the amount of the labeled antibody, the concentration of the capture antibody, and the position of the test line play a crucial role in developing effective LFIA devices [22,23]. Typical sandwich-type immunoassays rely on the use of two antibodies, which specifically target different epitopes of the antigen. In the eventuality that the pathogen shows repeated copies of the same antigen or that the antigen is composed of repeated molecular domains, a single antibody targeting the repeated epitope can be employed successfully to develop a sandwich-type immunoassay. In a previous work, we evidenced a different behavior between resorting to a “single epitope” (SE) targeting sandwich assay, i.e., one antibody for capture and detection, and using a “double epitope” (DE) targeting sandwich assay, where the capture and detection antibodies differed [23]. Indeed, we observed that for DE assays, the increase in the antibodies led to an increase in the analytical signal. For the SE assays, the observed effect was the opposite; i.e., when increasing the amount of the antibodies, the signal decreased. In this work, we deeply investigated the phenomenon to possibly establish if the evidence could be generalized or if it was due to that specific antigen (e.g., to the particular number and distancing of the epitopes). Therefore, two lateral flow devices were designed, using gold nanoparticles (AuNPs) as the optical signal reporter (Figure 1) and passive adsorption for antibody attachment. Although covalent coupling of antibodies to AuNPs ensures the best orientation of the ligand for binding to the antigen [24], the adsorption strategy is universally employed due to its simplicity, cost efficiency, and preservation of antibody structure and binding ability [24,25]. One system employed a traditional sandwich-type immunoassay, in which two antibodies, which recognize two different epitopes of the LSDV p32, were used (DE, Figure 1b). To this end, these two mAbs (identified as #2C6 and #2F10) were either linked to the AuNPs or immobilized onto the membrane to explore their performance as capture and detection ligands. The optimal capture–detection combination was defined according to the stability of the resulting mAb_AuNP conjugate and to the highest.

Signal-to-noise ratio (i.e., the ratio between the color developed by applying the supernatant of an inactivated virus suspension and the color developed by applying the sample diluent). The second device was designed by using each of the mAb as the capture and detection ligand in combination with itself (SE, Figure 1a). We studied the effect of varying the characteristics of the mAb_AuNP probes (such as varying the optical density of the probe, the mAb-to-AuNP ratio, and the size of AuNPs) on assay sensitivity. The probes were characterized by UV-visible spectroscopy, zeta-potential, and high-resolution transmission electron microscopy (HR-TEM), and their effectiveness in the LFIA was studied by a full-factorial design of experiment (FF-DoE), mapping the intensity of the color obtained by a “positive control” (inactivated LSDV Neethling strain). Experiments were replicated for the SE system, using one mAb as the capture and detection ligand, and for the DE system, which employed the two mAbs. The two systems were optimized and compared to confirm whether the atypical hook effect, due to the saturation of antigen epitopes by the detection ligand, could be generalized and occurs for sandwich-type LFIA based on one mAb. The DE-based assay, which showed the best performance, was further characterized by evaluating the analytical sensitivity, the inter- and intra-assay repeatability, and the specificity towards other viruses of the *lentivirus* and *parapoxvirus* genii.

## 2. Materials and Methods

### 2.1. Synthesis and Characterization of the Gold Nanoparticles (AuNPs)

AuNPs with different sizes (24, 30, 36 nm) were prepared by tetra chloroauric acid reduction with different amounts of sodium citrate as previously described [26]. The colloidal gold solutions were spectroscopically characterized by means of a Cary 60 UV-Visible spectrophotometer (Agilent, Santa Clara, CA, USA) in the UV-visible range, and hydrodynamic diameters were acquired by using a Z-view^R^ Nanoparticle Tracking Analyzer PMX 120 (Particle Metrix Gmbh, Inning, Germany). The 32 nm AuNPs and conjugates were also characterized by high-resolution transmission electron microscopy (HR-TEM) (Jeol JEM 3010-UHR, Tokyo, Japan). 

### 2.2. Synthesis and Characterization of the mAb_AuNP Conjugates

Signal reporters used in the LFIAs were prepared by passively adsorbing the mAbs onto the surface of the AuNPs, followed by overcoating of the AuNP-free surface with BSA [24]. In detail, the pH of 10 mL of AuNP solution was adjusted to 8 with carbonate buffer (0.05 M, pH 9.6) and added to 1 mL of borate buffer (0.02 M, pH 8). The mAb solution was gently mixed with the AuNPs and incubated for 40 min at 37 °C at room temperature. Then, 1 mL of BSA (1% *w*/*v* in borate buffer) was added and reacted for 10 min. The mAb_AuNP conjugates were recovered by centrifugation (10,000 rpm, 15 min) and washed twice with borate buffer supplemented with 0.1% *w*/*v* BSA. Finally, mAb_AuNPs were re-suspended in AuNP storage buffer (borate buffer with 1% *w*/*v* BSA, 0.25% *v*/*v* Tween 20, *w*/*v* 2% sucrose, and 0.02% *w*/*v* sodium azide) and stored at 4 °C until use. The #2F10_AuNP conjugates made from the 32 nm AuNPs were characterized by their visible spectra], Z-potential, and dynamic diameter, and HR-TEM imaging. For these experiments, the mAb_AuNP were not overcoated with BSA. 

### 2.3. Production of the LFIA Strips

The capture mAbs (#2F10 and #2C6) used for drawing test lines and the Streptococcal protein G for the control line of the LFIA devices were diluted in phosphate buffer (0.02 M pH 7.4) and applied at 1 µL/cm onto the nitrocellulose membrane by means of a XYZ3050 platform (Biodot, Irvine, CA, USA) equipped with a BioJetQuanti 3000 Line Dispenser for non-contact dispensing. The mAb concentration used for the experimental design and in the final device was 1 mg/mL and 2 mg/mL, respectively. Strips were composed as follows: sample pad, conjugate pad, membrane, and adsorbent pad and were cut to 4.2 mm in width) by means of a CM4000 guillotine (Biodot, Irvine, CA, USA). Finally, strips were inserted into plastic cassettes (Kinbio, Bejing, China) to fabricate the ready-to-use LFIA device. Cassettes were stored in the dark in plastic bags containing silica at room temperature until use.

### 2.4. Experimental Design for the SE and for the DE

Three AuNPs preparations differing in size were used to produce mAb_AuNP conjugates. For each AuNP preparation, we changed, independently, (i) 4 levels of optical density for the SE (Table 1) and 3 for the DE (Table 2), and (ii) 4 levels of the mAb-to-AuNP ratio. The combinations were tested in duplicate by using a positive and a negative sample, for a total of 336 experiments. The probes were diluted with the AuNP dilution buffer (borate buffer with 0.25% Tween 20 *v*/*v*, 2% *w*/*v* sucrose and 0.02% *w*/*v* sodium azide) and adsorbed onto a pre-saturated glass fiber conjugate pad. Pads were dried for 4 h at room temperature. The format and condition giving the highest color intensity of the test line was extracted from the FF-DoE and the optimized device (LSD_LFIA). 

### 2.5. Viruses

The virulent LSD virus, the Neethling strain, was grown in the ovine testis cell line (OA3.Ts). The supernatant of infected cells was harvested when the cytopathic effect was at its maximum and combined with the supernatant recovered from cell debris subjected to freeze–thaw cycles. The infectious virus titer was expressed as Tissue Culture Infective Dose 50 (TCID50) calculated by the Reed–Muench method [27]. For inactivation, the virus suspension was clarified by centrifugation at 5000 rpm for 20 min, followed by filtration through 0.2 μm filters, and then β-Propiolactone was added at a concentration of 0.01% and kept for 48 h at 4 °C. Inactivation was confirmed by repeated passages in the OA3.Ts cell line. A series of virus isolates belonging to the family Poxviridae, as detailed in Table 3, were additionally used for LSD-LFIA initial validation. The sole running buffer was used as the negative control.

### 2.6. In-House Validation of the LSD-LFIA Device

The visual cut-off was defined as the LSDV level corresponding to the complete disappearance of the test line (visual LOD, vLOD). The specificity was evaluated by testing viruses belonging to the Poxviridae family, which LSDV also belongs to, and some viruses infecting bovine. The LSD-LFIA imprecision was estimated by measuring between- and within-assay reproducibility and was calculated by analyzing three dilutions of the inactivated virus suspension (4×, 1×, and 0.5× of the vLOD) in three replicates each, three times within the same day, and on three days. The overall assay variability was calculated as the mean of coefficients of variation (CV%) from all sessions for each level (*n* = 18). The within-assay variability was estimated based on the mean of the CV% within days (*n* = 9) and between days (*n* = 3). The shelf life of the device at 4 °C, room temperature, and 37 °C was explored after 7, 14, 30, and 90 days. The analytical sensitivity was evaluated in parallel with a sandwich ELISA using live LSDV Neethling strain growth on the ovine testis cell line (OA3.Ts), titrated by the Reed–Muench method, expressed as 50% Tissue Culture Infective Dose (TCID50), and tested in serial dilutions [28]. For the sandwich ELISA, the two selected mAbs (#2F10 and #2C6) were preliminarily evaluated for their capability in different combinations of adsorbed and HRP-conjugated MAb, to efficiently bind and detect the LSDV antigen [28]; thus, the combination 2F10 as adsorbed mAb and #2C6 as HRPO-conjugated mAb was selected. The 2F10 mAb was adsorbed to the microtiter plates (NUNC, Maxisorp, Roskilde, Denmark) at a saturating concentration of 2 µg/mL in a carbonate–bicarbonate buffer (pH 9.6), and after overnight incubation at 4 °C, three washes with PBS containing 0.05% Tween 20 were performed. The viral antigen was incubated for 1 h at 37 °C, at three-fold serial dilution. After three washes with the same buffer, the #2C6 HRP-conjugated mAb was added at serial checkerboard dilutions with respect to the viral antigen, for a further 1 h of incubation at 37 °C. Following three final washes with the same PBS–Tween buffer, 0.5 mg/mL of OPD diluted in phosphate–citrate buffer (pH 5.6) and supplemented with 0.02% H_2_O_2_ was added for 10 min of incubation at room temperature; the reaction was stopped with H_2_SO_4_ (1 M). Plates were analyzed using a Multiscan Ascent spectrophotometer (Thermo Fisher Scientific, Waltham, MA, USA) at 492 nm wavelength. The results are expressed as net optical density (OD) obtained by the OD of the reaction with the viral antigen subtracted by the OD value of the reaction without the viral antigen.

## 3. Results and Discussion

### 3.1. Preparation and Spectroscopic Characterization of the mAb_AuNP Conjugates

AuNPs with different localized surface plasmon resonance (LSPR) maximum wavelengths (523.0, 525.5, 527.0 nm) were prepared by reducing tetra chloroauric acid with different amounts of trisodium citrate. According to the model proposed by Khlebetsov et al., the mean diameters of the AuNPs were calculated as 24, 30, and 36 nm, respectively [29]. The amount of the antibody needed to stabilize AuNPs was defined by a salt-induced aggregation test and was identified as the one providing a 540/620 absorbance ratio (corresponding to the non-aggregated/aggregated nanoparticles) over 3, which represents the initial value shown by the unperturbed colloidal gold suspension. The salt-induced aggregation tests showed the typical behavior, with an increasing stabilization due to the increasing quantity of the antibody, which passivated the surface of the AuNPs and protected them from the aggregation (Figure S1). Interestingly, although the AuNPs involved in the study differed in size, the quantity of antibodies needed for the stabilization was apparently the same. The smallest AuNPs showed lower absorbance, and largest AuNPs were more prone to aggregation. As the 32 nm AuNPs are the typically employed probes in the LFIA, we continued the study on these AuNPs. The minimum amount of mAb required for stabilizing 1 mL of AuNP diluted at a level giving OD1 was defined as 6 µg for the mAb #2C6, and 10 µg for the mAb #2F10. 

However, with the aim of maximizing the sensitivity of the LFIA for LSD diagnosis and, concurrently, to investigate the effect of some key parameters on assay performance, we synthesized several #2F10-AuNP conjugates by varying the mAb-to-AuNP ratio as a fraction or a multiple of the stabilizing amount (Figure S2a). The #2F10_AuNP conjugates made from the 32 nm AuNPs were also characterized by their visible spectra, Z-potential, and hydrodynamic diameter (Table 2), and HR-TEM imaging (Figure S2b–f). The Vis spectra in Figure S2g and their first derivative (Figure S2h) show a slight blue shift of the maximum of the LSPR corresponding to the addition of 10 µg, 15 µg, and 20 µg of the mAb. On the contrary, the addition of 5 µg caused a modification of the peak profile, which can be ascribed to the conjugate instability and aggregation phenomena. This is consistent with the flocculation stress test, in which the minimum quantity of the mAb needed for stabilization was set at 10 µg. The band shift of 4 nm observed upon the addition of 10 µg of mAb was consistent with the addition of a layer of bioreagents on the AuNP surface (Table 3). 

**Figure 2:**
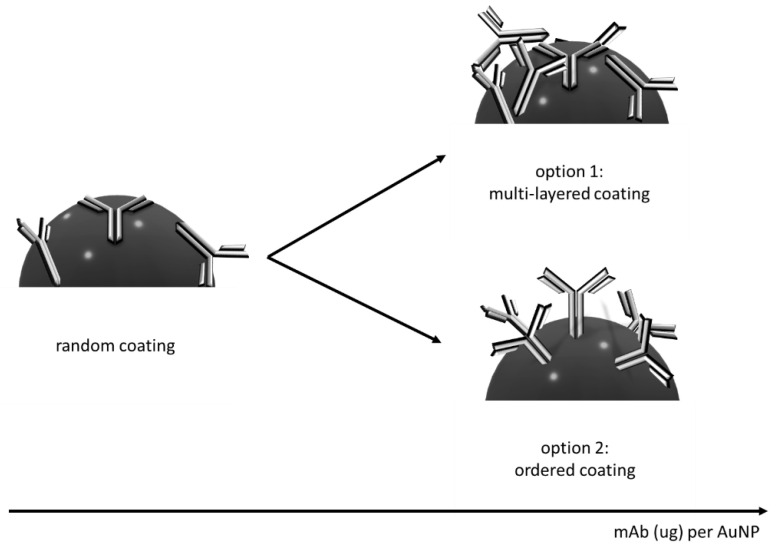
Scheme of the hypothesized increase in mAb adsorbed onto AuNP: (**1**) absorption of a second layer of randomly oriented mAb; (**2**) the additional mAb forced the head-on/tail-on orientation.

Interestingly, the addition of higher amounts of the mAb induced a further limited increase in the λ_max_ (0.5 nm each for 15 and 20 µg of the mAb, respectively). This result suggests that the quantity of mAb adsorbed onto the AuNP surface varied, though not to the extent that it was compatible with the formation of a second mAb layer. We hypothesized that increasing the mAb put into contact with AuNPs over the stabilizing value resulted in a larger amount of the bioligand adsorbed. This was also supported by the measurement of the hydrodynamic diameter and Z-potential (Table 3). The thickness of the bioligand layer adsorbed onto the AuNP was estimated as the difference between hydrodynamic diameters of the mAb_AuNP conjugate and the one of the pristine AuNP, divided by two [29]. According to the calculation, the protein layer was 2.1 nm-thick when the stabilizing amount of mAb was adsorbed and increased to 3.2 nm when the amount was doubled. Two possible phenomena can explain the increase: (1) the mAb added formed a second layer of bioligands with a random orientation, or (2) the addition of the mAb induced the formation of an increasingly bundled monolayer of the bioligand, forcing the orientation of the antibody towards head-on/tail-on, in such a way that the Fab was more exposed for the binding (Figure 2) [25]. 

Though both reasonable, only the second hypothesis was also able to explain the parallel increase in the binding ability with the increasing quantity of mAb adsorbed. All mAb_AuNP conjugates showed a largely negative z-potential, which varied from the −41.7 mV value of the bare AuNP to −24–29 mV for the conjugates prepared by adsorbing the stabilizing amount of the mAb or exceeding this value. The z-potential for these conjugates was comparable to the one of mAb at pH 8.0, thus confirming the formation of the antibody layer on the AuNP surface when 10 µg of the mAb was adsorbed [30]. Coherently with the use of an insufficient amount of antibody to cover the AuNP surface, the conjugate prepared by adsorbing 5 µg of the mAb showed a more negative z-potential between the one of citrate-capped AuNPs and the one corresponding to AuNP completely shielded by the mAb layer. According to the suggested aggregation, the 5 µg conjugate showed an apparent diameter of about 90 nm. 

### 3.2. Development of the LSDV_LFIA Based on the Combination #2F10_AuNP/#2F10 (SE)

**Figure 3:**
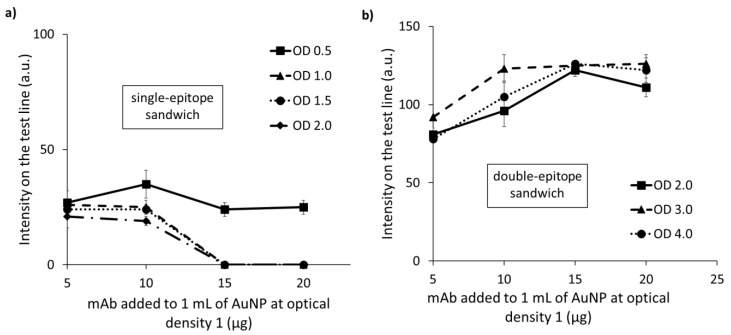
Intensity map for the 32 nm AuNPs with increasing amounts of #2F10_AuNP (optical density 0.5-1-1.5-2 for the SE (**a**) and 2-3-4 for the DE (**b**)) and antibody adsorbed to the surface of the AuNPs (5-10-15-20 µg for each mL of AuNP at optical density 1).

Combining two mAbs conjugated to AuNPs (detectors, #2F10* and #2C6*) and the same two used as capturing bioreagents (#2F10c and #2C6c), four combinations were possible and were investigated: two DEs (#2F10*/#2C6c, #2C6*/2F10c), and two SEs (#2F10*/#2F10c and #2C6*/#2C6c). Preliminarily, we excluded non-specific bindings in the explored combinations. DEs and SEs employing #2F10* showed the absence of non-specific signals, whereas the ones with #2C6* showed a strong non-specific signal. Some attempts were made to mitigate this non-specific binding by modifying: (i) the running buffer, (ii) the protein used to saturate the AuNP-free surface, and (iii) the mAb_AuNP storage buffer used for the dilution of the gold conjugate. In brief, we changed the type (BSA and casein) and concentration (1, 0.25, 0.1% *w*/*v*) of the protein and the ionic strength of the running buffer by adding different amounts (30, 80, 130, 180 mM) of sodium chloride. The casein was used for its ability to interfere with protein–protein non-specific interactions, while the ionic strength was used to reduce electrostatic interactions. The addition of 0.1% *w*/*v* casein and 80 mM sodium chloride to the running buffer reduced the non-specific binding when 6 µg of #2C6 per mL of AuNPs was adsorbed. However, it was ineffective for higher mAb-to-AuNP ratios (Table S1). As the attempts made to reduce non-specific interaction of the #2C6* were unfruitful, the combinations involving this probe were no further studied. The optimal conditions for detecting LSDV by the LFIA including the same mAbs as the capture and detection reagents were defined by exploiting an FF-DoE. This was made by mapping the intensity of the test line as a function of the mAb-to-AuNP ratio and optical density. As previously observed for a similar LFIA for FMDV detection, the SE showed a saturation behavior, where the intensity of the test line decreased by increasing the 2F10* optical density (Figure S3 and Table S2) [23]. The decrease in the signal, while increasing antigen concentration, occurs for sandwich-type immunoassays when the antigen amount exceeds the capture and/or detection antibodies. The effect, named the “hook effect”, has been explained as the overwhelming inhibition by antigen-saturated capture and detection antibodies of the formation of the capture–antigen–detection “sandwich” complex that is responsible for the antigen-related signal. To limit the hook effect and increase the dynamic range of the assay, typically, the quantity of the antibodies should be augmented. In the LFIA platform, the equilibria between the antigen and capture and detection antibodies occur separately, so the effect is just apparently similar to the typical hook effect. In this case, the observed decrease in the signal was not due to the antigen but to the excess of the detection antibody. The effect was described as an “antigen saturation hook effect” (asHE) (Figure 3a) where the capture and the detection antibodies competed for the same epitope of the antigen. 

In this competition, the detection antibody was favored considering that it was mixed with the antigen in the solution and that it had much more contact time with the antigen compared to the capture antibody. The saturation of the antigen by the detection antibody inhibited its further binding to the capture antibody at the test line and resulted in a lowering of the test line color. The same behavior happened when increasing both the quantity of mAb-AuNP and the mAb-to-AuNP ratio. The experimental design was repeated with AuNPs of various sizes. From the data, we always observed the asHE phenomenon, regardless of the particle size (Figure S3). 

### 3.3. Development of the LSDV_LFIA Based on the Combination #2F10_AuNP/#2C6 (DE)

When the DE was developed, we observed the opposite effect than that seen for the SE, and the pattern of color intensities followed the typical trend expected for non-competitive immunoassays: the intensity of the test line (reported as arbitrary units, a.u., from the image processing) increased with the increase in the antibody amount, both considered as the OD of the conjugate and the mAb-to-AuNP ratio parameters (Figure 3b). As for the SE, we completed the experimental design with various sizes of AuNPs (Figure S4 and Table S3). From the intensity maps, we determined the experimental conditions leading to the maximal signal for both the SE and DE approaches. The top performing condition for the SE approach was characterized by 36 nm AuNPs functionalized with 5 µg for each milliliter of AuNP of optical density 1, and used at an optical density of 0.5. As an alternative, the 32 nm AuNPs were also effective when functionalized with 10 µg for each milliliter of AuNP of optical density 1 and used at an optical density of 0.5. Both the conditions provided a signal between 35 and 40 a.u. from the digitalizing processing of the test line color for the inactivated virus suspension. Much more intense signals were achieved with the DE, for which the top performing condition was obtained from 32 nm AuNPs functionalized with 20 µg for each milliliter of AuNP of optical density 1 and used at an optical density of 3. This condition provided a signal of 127 a.u., overcoming the top performers of the SE approach by a factor of 3.6 and 3.2, respectively. Therefore, the optimized DE (32 nm, 20 µg/mL, OD3) was selected for the LSDV_LFIA development. 

### 3.4. The LFIA Device for the Rapid LSD Antigen Detection

Further refining of the assay sensitivity was realized by modulating additives used for AuNP conjugate preparation and for the running buffer, until reaching of a substantial gain in the color intensity (Table 3). In particular, by combining the use of a hydrogen carbonate buffer (26 mM pH 7.9 supplemented with 1% *v*/*v* tween 20 and 0.02% *w*/*v* sodium azide, without any additional proteins) as the running buffer, and the use of a borate buffer (20 mM pH 8 supplemented with 2% *w*/*v* sucrose, 0.25% *v*/*v* Tween 20, 0.02% *w*/*v* sodium azide, 0.8% *w*/*v* BSA, and 0.1% *w*/*v* casein) as the mAb_AuNP dilution buffer, we obtained an increment in the signal-to-noise ratio by a factor of three compared to the use of the typical phosphate buffer.

Furthermore, by doubling the concentration of the capture antibody, the signal increased again by a factor of about three (Table S5).

**Figure 4:**
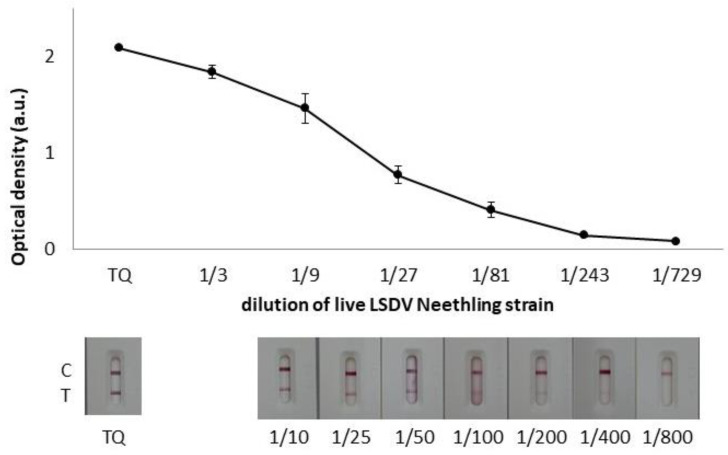
LSD_LFIA sensitivity assessment by testing serial dilution of live LSDV Neethling strain titrated as 10^6^ TCID_50_/mL in parallel with the ELISA.

The optimized LSDV-LFIA was assessed with serial dilutions of the live LSDV Neethling strain in parallel with the sandwich ELISA test (Figure 4). The test line was still visible at 400-fold dilution of the viral antigen. In the sandwich ELISA, the conditions with the best signal-to-noise ratio were considered, and the cut-off was set at 1/250 (net OD ranged between 0.1 and 0.15). In accordance with the virus titration of 10^6^ TCID_50_/mL, the visual limit of detection (vLOD) for the LSDV-LFIA resulted in 10^3.4^ TCID_50_/mL, which is comparable with the value obtained in the sandwich ELISA (10^3.6^ TCID_50_/mL). Although ELISA is an accurate and reproducible analytical method and it is largely employed for the diagnosis of infectious diseases, its applicability is confined to the laboratory, and it is not suited for timely and onsite testing. It is a general opinion that the LFIA possesses lower sensitivity, specificity, and higher variability in results than laboratory-based immunoassays, such as ELISA. In this work, we showed that the optimization of the LFIA allows for obtaining comparable performance, while the same antibodies are employed.

As for the imprecision assessment, we selected three levels corresponding to 4×, 1×, and 0.5× of the vLOD and repeated the analysis in triplicate in each experimental session (same sample preparation, same batch of strips) for three experimental sessions (new sample preparation, different strip batch) each day for three days. No false positives were reported considering the total number of experiments for the inactivated virus suspension diluted below the vLOD (*n* = 18). Moreover, no false-negative results were observed considering the experiments including the inactivated virus suspension at levels above the vLOD (*n* = 36). As summarized in Table 4, the assay showed very low CV% for both within- and between-assay variability for the 4x vLOD level (5.3% and 4.4%), which increased for the 1x vLOD level (20.3% and 26.2%). However, these values were still considered to be acceptable as they were measured in correspondence of the limit of detection of the method.

The device was evaluated for its specificity towards one lentivirus infecting bovine and viruses belonging to Poxviridae family, namely five orf virus isolates, four bovine popular stomatitis viruses, and two pseudocowpox viruses; no false-positive results were observed, proving that the assay was specific for the LSDV (Table 5).

The stability of the devices was verified after 90 days from the production by applying the running buffer and a positive sample (inactivated virus suspension) to LSD_LFIA devices stored at 4, 25, and 37 °C. No appreciable loss in color intensity was observed in any of the three conditions, and no false positive/false negatives were obtained.

## 4. Conclusions

We had previously observed a peculiar hook effect in the LFIA [23] and made the hypothesis that it was due to the antigen saturation caused by the excess of the labeled antibody. Therefore, we compared the cases of one antibody used as a capture and detector ligand (i.e., one epitope of the antigen is involved in the binding with both ligands, so that the saturation of the antigen by the detector impedes its subsequent capture by the reagent deposed on the test line) and two antibodies targeting different epitopes (where we expected that the two would not interfere reciprocally and the capture would not be hampered by the excess of the detector). We suggested that antigen saturation occurred in the time frame between detector resuspension by the sample and reaching the capture ligand (test line), which encompassed a few seconds. Although the contact time between the detector and the antigen was quite low, the large excess of the detector antibody generally used to increase the sensitivity of the sandwich-type LFIA caused the partial or complete saturation of the available epitopes of the antigen. In conclusion, the modulation of the reagents should be considered very carefully to balance detectability with saturation. In this work, we demonstrated that the variables influencing the saturation phenomena are different (AuNP size, mAb-to-AuNP ratio, optical density). To limit the number of experiments (and the consumption of precious reagents), we applied a design of experiment strategy to optimize the variables, instead of the traditional checkerboard titration, which allows for optimizing just two variables at one time. The signal intensity (and, therefore, assay sensitivity) was used to define optimal conditions.

To investigate the peculiar hook effect, we used a model case, i.e., the detection of the lumpy skin disease virus. By employing two monoclonal antibodies specific for LSDV, we traced the route for the optimization of the LFIA in the cases of using both one and two antibodies for the viral antigen detection. Indeed, even if using different antibodies is certainly more convenient and also proved to lead to higher sensitivity in our work, is not uncommon to have just one efficient antibody available (or to have more than one but it binds to the same or adjacent epitopes). As previously observed for the competitive LFIA, as well as for the sandwich LFIA employing one antibody, involving the competition between the capture and detector ligand, the best approach follows the rule “less is more” [31]: the lower the amount of the detector antibody, the higher the sensitivity. In particular, the effect of the optical density, and even more so, the mAb-to-AuNp ratio, should be low to provide better performances.

Moreover, to the best of our knowledge, no LFIA has been reported in the literature yet for the on-field rapid detection of the LSDV antigen. In the panorama of LSDV diagnosis, molecular methods are largely predominant, and immunoassays are used for serological indirect diagnosis and immunohistochemistry. We presented the very first lateral flow test for the diagnosis of LSDV antigen, which was confirmed to be as sensitive as the ELISA when exploiting the same antibody pair, reproducible, specific, and robust. The LSD_LFIA was confirmed to be as sensitive as the ELISA when exploiting the same antibody pair, reproducible, specific, and robust. So, the LSD_LFIA candidate itself has proved to be a useful tool for the on-field rapid diagnosis of LSD considering all the advantages of a rapid on-field testing method. The clinical validation of the assay through analyzing samples belonging to infected animals is ongoing and will reinforce its application for helping develop strategies to control LSD transmission and spread.

## Figures and Tables

**Table 1:** The FF-DoE scheme for SE optimization. Each experiment was repeated twice.

mAb-to-AuNP (× 10 µg/mL) ^a^	OD	Size of AuNP (nm)								
		24			32			36		
0.5×	0.5	−2	−2	−1	−2	−2	0	−2	−2	1
	1	−2	−1	−1	−2	−1	0	−2	−1	1
	1.5	−2	1	−1	−2	1	0	−2	1	1
	2	−2	2	−1	−2	2	0	−2	2	1
1.0×	0.5	−1	−2	−1	−1	−2	0	−1	−2	1
	1	−1	−1	−1	−1	−1	0	−1	−1	1
	1.5	−1	1	−1	−1	1	0	−1	1	1
	2	−1	2	−1	−1	2	0	−1	2	1
1.5×	0.5	1	−2	−1	1	−2	0	1	−2	1
	1	1	−1	−1	1	−1	0	1	−1	1
	1.5	1	1	−1	1	1	0	1	1	1
	2	1	2	−1	1	2	0	1	2	1
2.0×	0.5	2	−2	−1	2	−2	0	2	−2	1
	1	2	−1	−1	2	−1	0	2	−1	1
	1.5	2	1	−1	2	1	0	2	1	1
	2	2	2	−1	2	2	0	2	2	1

^a^ Defined as a n-fold multiple of the minimum stabilizing mAb-to-AuNP ratio.

**Table 2:** The FF-DoE scheme for DE optimization. Each experiment was repeated twice.

mAb-to-AuNP (× 10 µg/mL) ^a^	OD	Size of AuNP (nm)								
		24			32			36		
0.5×	2	−2	−1	−1	−2	−1	0	−2	−1	1
	3	−2	0	−1	−2	0	0	−2	0	1
	4	−2	1	−1	−2	1	0	−2	1	1
1.0×	2	−1	−1	−1	−1	−1	0	−1	−1	1
	3	−1	0	−1	−1	0	0	−1	0	1
	4	−1	1	−1	−1	1	0	−1	1	1
1.5×	2	1	−1	−1	1	−1	0	1	−1	1
	3	1	0	−1	1	0	0	1	0	1
	4	1	1	−1	1	1	0	1	1	1
2.0×	2	2	−1	−1	2	−1	0	2	−1	1
	3	2	0	−1	2	0	0	2	0	1
	4	2	1	−1	2	1	0	2	1	1

^a^ Defined as a n-fold multiple of the minimum stabilizing mAb-to-AuNP ratio.

**Table 3:** Spectroscopic and dimensional characteristics of conjugates as a function of different amounts of mAb adsorbed on the surface of the 32 nm AuNP.

Amount of mAb for mL of AuNP (OD1)	λmax of LSPR	Hydrodynamic Diameter ^a^	∆ ^b^	Z-Potential
(µg)	(nm)	(nm)	(nm)	(mV)
0	525.5	46.4 ± 0.3		−41.7 ± 0.4
5	536.0	89.8 ± 3.8	(21.7) ^c^	−34.5 ± 0.7
10	529.5	50.6 ± 1.5	2.1	−24.2 ± 0.3
15	530.0	51.6 ± 0.2	2.6	−23.5 ± 0.5
20	530.5	52.7 ± 1.7	3.2	−29.7 ± 1.2

^a^ as measured by the Z-view^R^ Nanoparticle Tracking Analyzer. ^b^ (hydrodynamic diameter of the conjugate—hydrodynamic diameter of the bare AuNP)/2. ^c^ the value represents the effect of aggregation rather than that of mAb adsorption.

**Table 4:** Imprecision of the LFD-LFIA.

	Within-Assay			Between-Assay		
**Level**	**Within-Day (*n* = 3)**	**Between-Day (*n* = 3)**	**Overall (*n* = 9)**	**Within-Day (*n* = 3)**	**Between-Day (*n* = 3)**	**Overall ^a^ (*n* = 9)**
2× vLOD	3, 7	7, 1	5, 3	0, 6	8, 3	4, 4
1× vLOD	19, 1	21,2	20, 3	27, 5	25, 7	26,2

^a^ calculated as mean coefficient of variation % (CV%).

**Table 5:** List of the viruses tested to evaluate the specificity of LSDV_LFIA.

Sample (#)	Genus	Species	Sampling (m/y)	LSD-LFIA
1	lentivirus	…	…	NEG
2	parapoxvirus	Orf virus	…	NEG
3		Orf virus	…	NEG
4		Orf virus	…	NEG
5		Orf virus	…	NEG
6		Orf virus	…	NEG
7		Bovine papular stomatitis virus	8/2008	NEG
8		Bovine papular stomatitis virus	2/2008	NEG
9		Bovine papular stomatitis virus	8/1998	NEG
10		Bovine papular stomatitis virus	4/2020	NEG
11		Pseudocowpox	5/2021	NEG
12		Pseudocowpox	5/2009	NEG

## Data Availability

Not applicable.

## Supplementary Materials

The following supporting information can be downloaded at: https://www.mdpi.com/article/10.3390/bios12090739/s1. Chemicals, AuNP synthesis, flocculation test [32]. Figure S1: Flocculation stress test for the mAb #2C6 (a) and for the mAb #2F10 (b). The ratio between Absorbance at 540 nm and Absorbance at 620 nm versus the amount of mAb used for each millilitre of AuNP with different size of AuNPs is plotted. The stabilizing amounts of the mAbs #2C6 and #2F10 are highlighted with a circle. Figure S2: Characterization of the 32 nm AuNPs and its conjugates with #2F10 mAb. (a) The flocculation stress test carried out by adsorbing 2–4–6–8–10 μg of the mAb to 1 mL of AuNP (OD1) followed by saline shock with aqueous 10% w/v NaCl: the absorbance at 540 nm and 620 nm are due to non-aggregated and aggregated fractions of AuNP, so increasing the stabilization increases the 540/620 absorbance ratio; (b–f) HR-TEM images of the mAb_AuNP from figure 2a, (g) visible spectra of the AuNP and conjugates obtained by increasing the amount of mAb adsorbed (0–5–10–15–20 μg per ml of AuNP) and (h) their first derivative. Figure S3: Response surface from the FF-DoE for the SE based on the mAb #2F10 and using different sizes of AuNP: 24nm (a), 32 nm (b), and 36 nm (c). Data were processed by means of the Software CAT. Figure S4: Response surface from the FF-DoE for the DE based on the mAb #2F10 as the detection and #2C6 as the capture ligand and using different sizes of AuNP: 24 nm (a), 32 nm (b), and 36 nm (c). Data were processed by means of the Software CAT. Table S1: Attempts to eliminate the background signal observed when using the #2C6_AuNP gold conjugate the mAb-to-AuNP ratio was 12 μg (2.0×) per mL of AuNP and the probe was diluted to optical density equal to 4. A solution of the inactivated viral culture (+) and the sample diluent (−) were applied and the colour intensity at the test line quantified. The condition selected is highlighted in bold. Table S2: Results from the design of experiment for the optimization of the SE based on the mAb #2F10. A 1 + 3 dilution solution of the inactivated viral culture in the sample diluent was used as the positive control and the colour intensity at the test line quantified. Table S3: Results from the design of experiment made on the DE based on the mAb #2F10 as the detection and #2C6 as the capture ligand. A 1 + 3 dilution solution of the inactivated viral culture in the sample diluent was used as the positive control and the colour intensity at the test line quantified. Table S4: Effects of the composition of the buffer used as running buffer and #2F10_AuNP dilution buffer. Colour of the test line was measured upon application of a solution of the inactivated viral culture (+) and of the running buffer (−). The condition selected is highlighted in bold. Table S5: Study of the optical density of the #2F10-AuNP probe (mAb-to-AuNP 20 μg per mL) for the device including the test line at 2 mg/mL. The condition selected is highlighted in bold.
