# Supplementary material for: Investigation of the “Antigen Hook Effect” in Lateral Flow Sandwich Immunoassay: The Case of Lumpy Skin Disease Virus Detection
Source: Biosensors (Basel). 2022 Sep 8;12(9):739. doi: 10.3390/bios12090739 (PMC9496205; doi:10.3390/bios12090739)
Supplement: Supplementary file 1 [file biosensors-12-00739-s001.zip › biosensors-1865991-supplementary.pdf]

SUPPLEMENTARY INFORMATION

Investigation of the "antigen hook effect" in lateral flow sandwich immunoassay: the case of lumpy skin disease virus detection

Simone Cavalera[1], Giulia Pezzoni[2], Santina Grazioli[2], Emiliana Brocchi[2], Stefano Baselli[2], Davide Lelli[2], Barbara Colitti[3], Thea Serra[1], Fabio Di Nardo[1], Matteo Chiarello[1], Valentina Testa[1], Sergio Rosati[3], Claudio Baggiani[1] and Laura Anfossi[1*]

[1] Department of Chemistry, University of Turin, Turin, Italy

[2] Istituto Zooprofilattico Sperimentale della Lombardia e dell'Emilia-Romagna, National/OIE/FAO Reference Centre for FMD and SVD, Brescia, Italy

[3] Department of Veterinary Science, University of Turin, Turin, Italy

Chemicals

Gold (III) chloride trihydrate (ACS reagent), sucrose, and bovine serum albumin (BSA) were obtained from Sigma–Aldrich (St. Louis, MO, USA). The anti-LSD mAbs (#2C6, #2F10) and the LSD virus were produced at IZSLER. Tween20 and other chemicals were purchased from VWR International (Milan, Italy). Nitro-cellulose membranes with cellulose adsorbent pad and blood separator sample pads were purchased by MDI membrane technologies (Ambala, India) and glass fibre conjugate pads were obtained from Merck Millipore (Billerica, MA, USA).

AuNP synthesis

AuNPs with different sizes (24, 30, 36 nm) were prepared by reducing $2.94x10^{-5}$ mol of tetrachloroauric acid with different amounts ($4.59x10^{-6}$, $4.93x10^{-6}$, $5.27x10^{-6}$ mol) of trisodium citrate dihydrate in boiling milliQ water refluxing with a six-bulb condenser. The localised surface plasmon resonance (LSPR) maximum wavelengths (523.0, 525.5, 527.0 nm) were acquired by means of a Cary 60 UV-Visible spectrophotometer (Agilent, CA, USA).

Flocculation stress test

To define the amount of mAb that stabilizes AuNP, the flocculation stress test was applied [1]. Therefore, 250 µl of AuNP solution at optical density 1 were inserted in wells of a microtiter plate and incubated for 30 min with increasing amounts (0-2.5µg) of the mAb. Then, 25 µl of aqueous NaCl (10% v/v) were added and reacted for 10 min to promote aggregation of unstable AuNPs. The absorbance of the solutions was read at 540 and 620 nm by a microplate reader (Multiskan FC, Microplate Photometer). Results of the flocculation stress test for AuNP with mean diameters of 24, 32, and 36 nm are shown in Figure S1a and b for the mAb #2C6 and #2F10, respectively. The stabilizing amount of mAb was approximately the same, regardless of the AuNP dimension. Instead, the two mAbs showed a different ability to protect AuNP from aggregation, as 6µg of the #2C6 mAb were sufficient to stabilize gold colloids, while 10µg of the #2F10 were needed.

TABLES

Table S1: Attempts to eliminate the background signal observed when using the #2C6_AuNP gold conjugate the mAb-to-AuNP ratio was 12µg (2.0x) per mL of AuNP and the probe was diluted to optical density equal to 4. A solution of the inactivated viral culture (+) and the sample diluent (-) were applied and the colour intensity at the test line quantified. The condition selected is highlighted in bold.

Sample diluent [a]	Overcoating [b]	mAb_AuNP Dilution buffer [c]	(-)	(+) [d]
1% BSA	0.1% w/v BSA	1% w/v BSA	460 ± 12	470 ± 40
0.25% w/v casein	0.5% w/v casein		54 ± 5	91 ± 4
		0.5% w/v casein	8 ± 3	17 ± 3
	0.1% w/v BSA	1% w/v BSA	122 ± 8	75 ± 15
0.1% w/v casein + 30mM NaCl	0.1% w/v BSA		24 ± 3	49 ± 2
**0.1% w/v casein + 80mM NaCl**	**0.1% w/v BSA**	**1% w/v BSA**	**nd**	**95 ± 14**
0.1% w/v casein + 130mM NaCl			nd	96 ± 9
0.1% w/v casein + 180mM NaCl			nd	99 ± 13

[a] Hydrogencarbonate buffer 26mM pH 7.9 + 1% tween20 + 0.02% w/v sodium azide

[b] Borate buffer 20mM pH 8

[c] Borate buffer 20mM pH 8 + 2% w/v sucrose + 0.25% v/v Tween20 + 0.02% w/v sodium azide

[d] 1+1 running buffer diluted inactivated viral suspension

Table S2: Results from the design of experiment for the optimization of the SE based on the mAb #2F10. A 1+3 dilution solution of the inactivated viral culture in the sample diluent was used as the positive control and the colour intensity at the test line quantified.

AuNP size (nm)	OD	mAb-to-AuNP (x 10µg/mL)[a]			
		0.5x	1.0x	1.5x	2x
24	0.5	28 ± 0	32 ± 10	nd[b]	nd[b]
	1.0	nd[b]	nd[b]	nd[b]	nd[b]
	1.5	nd[b]	nd[b]	nd[b]	nd[b]
	2.0	nd[b]	nd[b]	nd[b]	nd[b]
32	0.5	27 ± 5	35 ± 6	24 ± 3	25 ± 3
	1.0	26 ± 0	25 ± 3	nd[b]	nd[b]
	1.5	24 ± 4	24 ± 2	d[b]	nd[b]
	2.0	21 ± 5	19 ± 2	nd[b]	nd[b]
36	0.5	39 ± 15	29 ± 2	30 ± 2	31 ± 5
	1.0	33 ± 11	26 ± 6	23 ± 1	20 ± 2
	1.5	31 ± 5	26 ± 3	nd[b]	nd[b]
	2.0	21 ± 1	15 ± 3	nd[b]	nd[b]

[a] Defined as a n-fold multiple of the minimum stabilising mAb-to-AuNP ratio

[b] Not detectable by the software QuantiScan

Table S3: Results from the design of experiment made on the DE based on the mAb #2F10 as the detection and #2C6 as the capture ligand. A 1+3 dilution solution of the inactivated viral culture in the sample diluent was used as the positive control and the colour intensity at the test line quantified.

AuNP size (nm)	OD	mAb-to-AuNP (x 10µg/mL)[a]											
		0.5x			1.0x			1.5x			2.0x		
24	2	71	±	1	52	±	6	45	±	8	35	±	0
	3	61	±	2	68	±	7	36	±	1	37	±	2
	4	59	±	13	64	±	14	53	±	4	47	±	5
32	2	81	±	4	96	±	10	122	±	4	111	±	6
	3	92	±	2	123	±	9	125	±	3	126	±	6
	4	78	±	2	105	±	10	126	±	2	122	±	8
36	2	95	±	7	80	±	3	108	±	5	126	±	17
	3	98	±	3	84	±	11	108	±	19	111	±	3
	4	93	±	3	89	±	9	116	±	12	127	±	18

[a] Defined as a n-fold multiple of the minimum stabilising mAb-to-AuNP ratio

Table S4: Effects of the composition of the buffer used as running buffer and #2F10_AuNP dilution buffer. Colour of the test line was measured upon application of a solution of the inactivated viral culture (+) and of the running buffer (-). The condition selected is highlighted in bold.

Running buffer	Protein	mAb_AuNP dilution buffer[c]	(-)			(+)		
TG[a]	0.2% w/v casein	1% w/v BSA	nd			30	±	4
	1% w/v BSA	1% w/v BSA	32	±	**3**	90	±	**7**
**HCB[b]**	1% w/v BSA	1% w/v BSA	38	±	6	104	±	14
	0.25% casein + 1%w/v BSA	1% w/v BSA	26	±	2	86	±	8
	0.25% w/v casein	1% w/v BSA	nd			44	±	0
	0.1% w/v casein	1% w/v BSA	17	±	1	57	±	4
	**no protein**	1% w/v BSA	30	±	12	112	±	5
	**no protein**	**0.8% w/v BSA + 0.1% w/v casein**	**nd**			**99**	**±**	**2**

[a] Tris-Glycin buffer 115mM pH 8.2 + 1% v/v tween20 + 0.02% w/v Sodium azide

[b] Hydrogen carbonate buffer 26mM pH 7.9 + 1% v/v tween20 + 0.02% w/v Sodium azide

[c] Borate buffer 20mM pH 8 + 2% w/v sucrose + 0.25% v/v Tween20 + 0.02% w/v sodium azide

Table S5: Study of the optical density of the #2F10-AuNP probe (mAb-to-AuNP 20µg per mL) for the device including the test line at 2mg/mL. The condition selected is highlighted in bold.

OD	Intensity of the colour of the test line
2	224
**3**	**254**

4	239
5	242

FIGURES

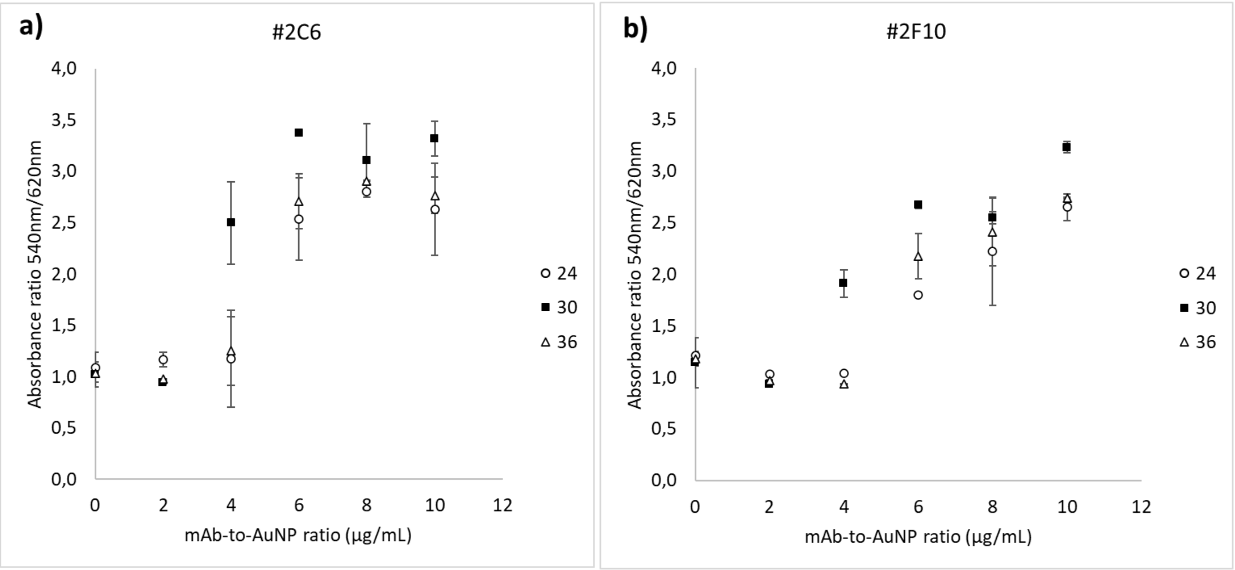

Figure S1: Flocculation stress test for the mAb #2C6 (a) and for the mAb #2F10 (b). The ratio between Absorbance at 540 nm and Absorbance at 620 nm versus the amount of mAb used for each millilitre of AuNP with different size of AuNPs is plotted. The stabilizing amounts of the mAbs #2C6 and #2F10 are highlighted with a circle.

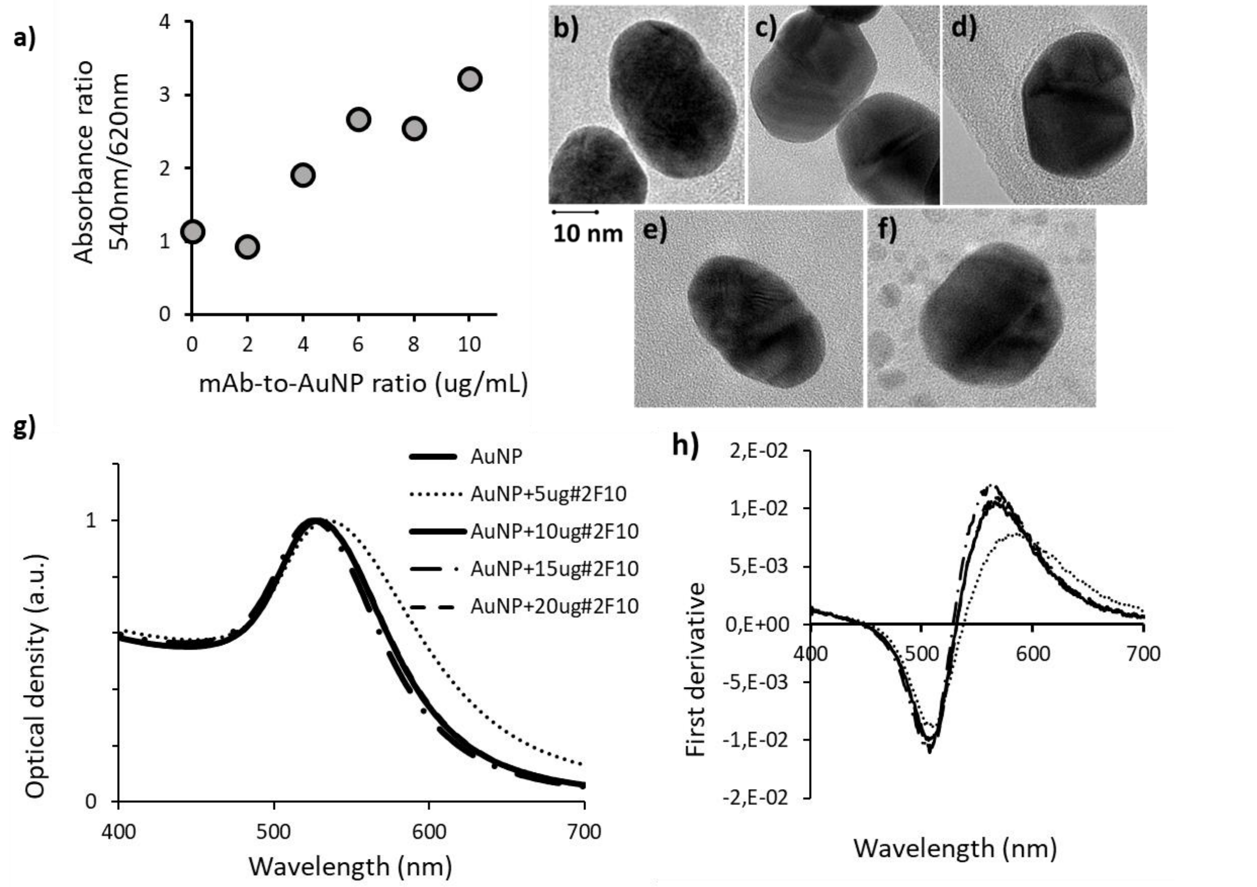

Figure S2: Characterization of the 32 nm AuNPs and its conjugates with #2F10 mAb. a) The flocculation stress test carried out by adsorbing 2-4-6-8-10µg of the mAb to 1 mL of AuNP (OD1) followed by saline shock with aqueous 10% w/v NaCl: the absorbance at 540 nm and 620 nm are due to non-aggregated and

aggregated fractions of AuNP, so increasing the stabilization increases the 540/620 absorbance ratio; b-f) HR-TEM images of the mAb_AuNP from figure 2a, g) visible spectra of the AuNP and conjugates obtained by increasing the amount of mAb adsorbed (0-5-10-15-20µg per ml of AuNP) and h) their first derivative.

a)

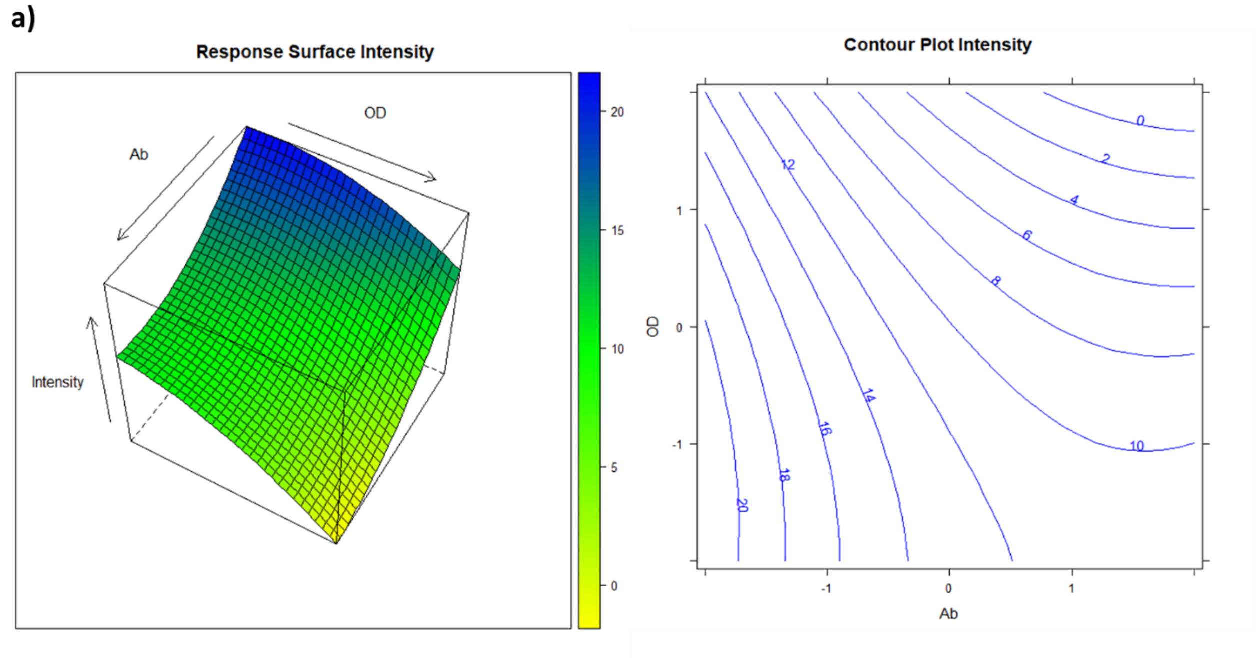

b)

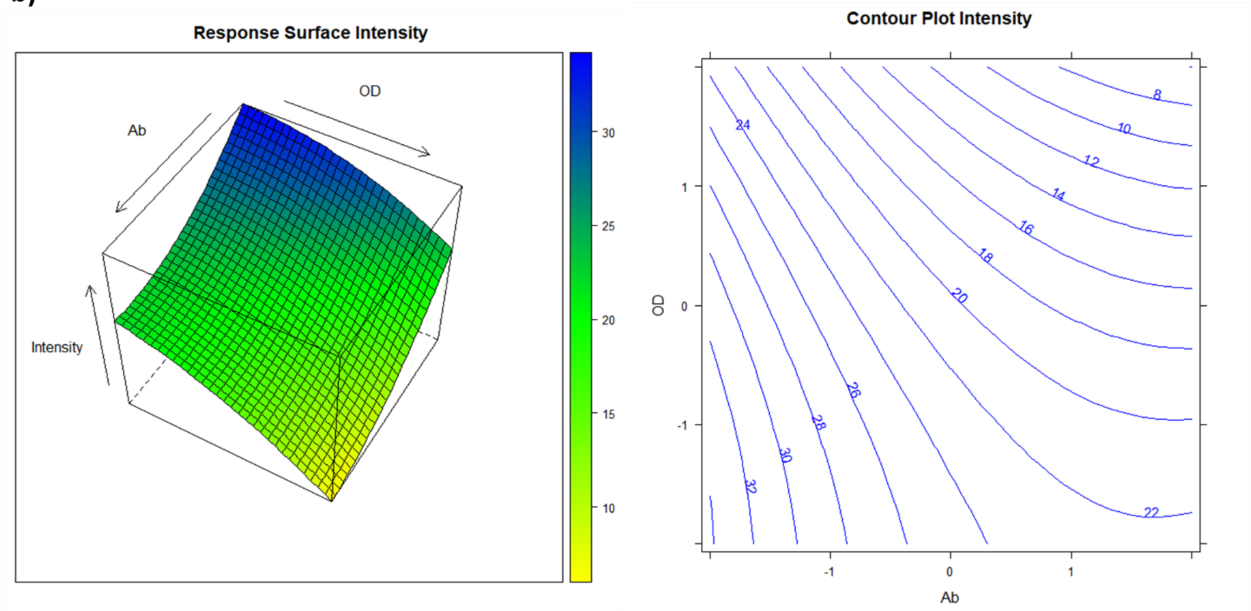

c)

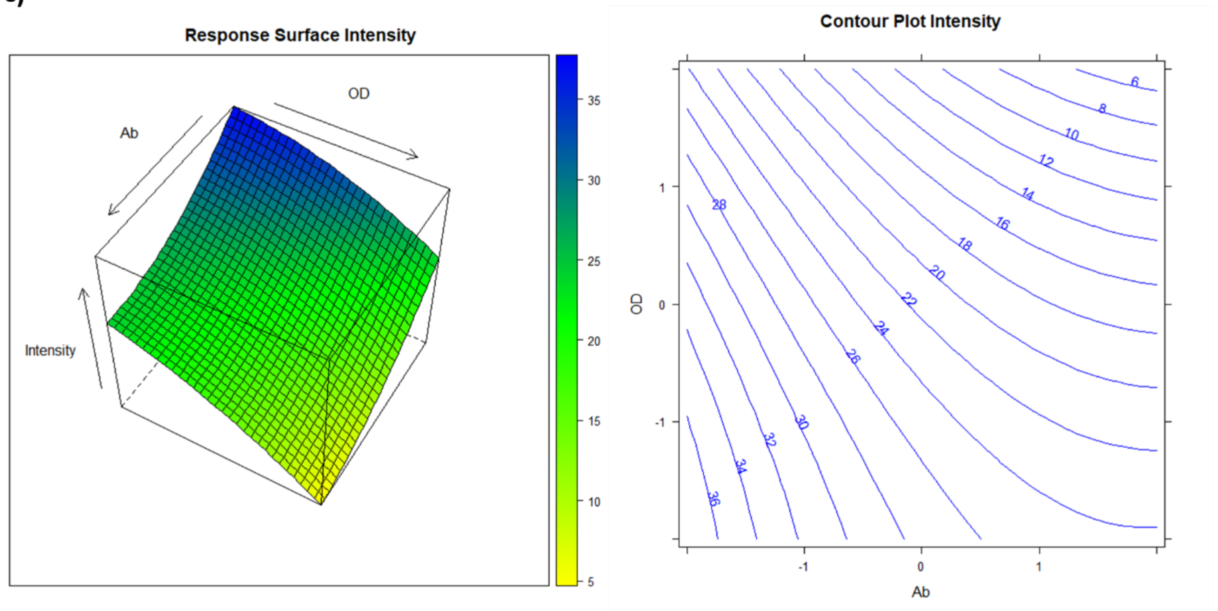

Figure S3: Response surface from the FF-DoE for the SE based on the mAb #2F10 and using different sizes of AuNP: 24nm (a), 32 nm (b), and 36nm (c). Data were processed by means of the Software CAT.

a)

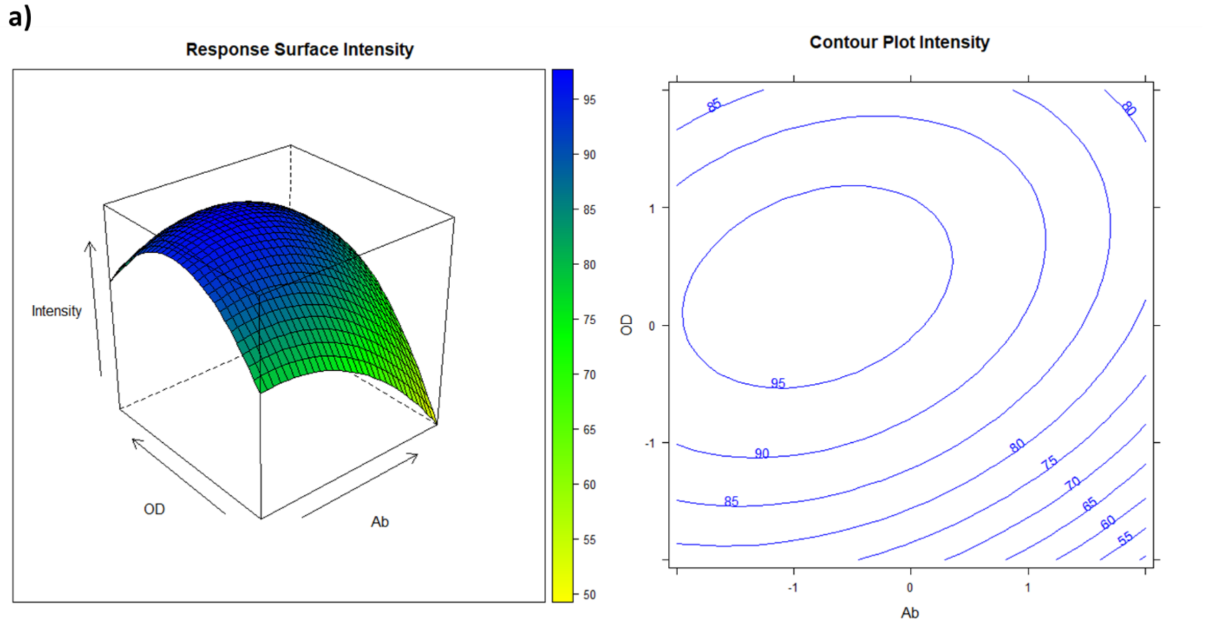

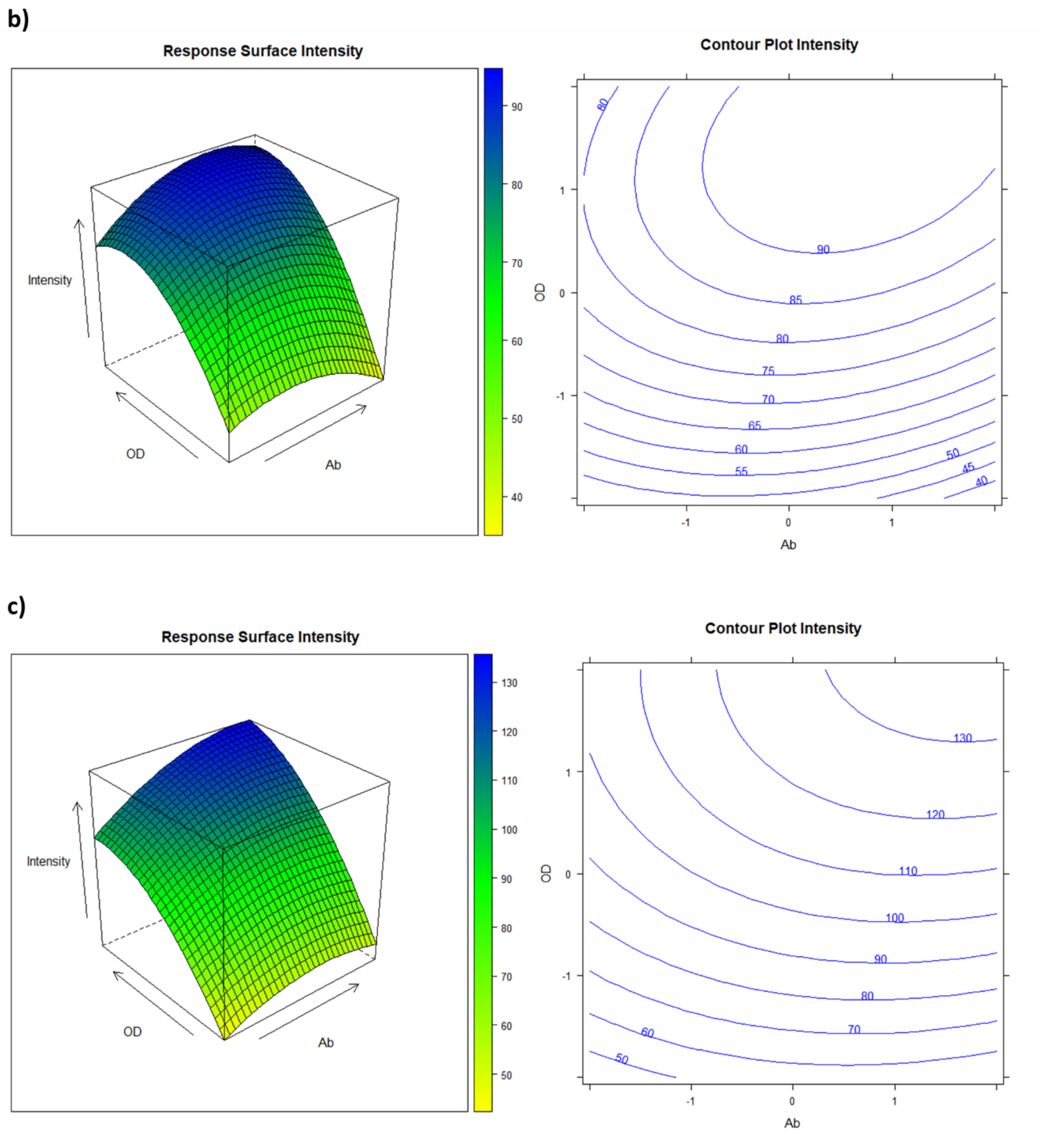

Figure S4: Response surface from the FF-DoE for the DE based on the mAb #2F10 as the detection and #2C6 as the capture ligand and using different sizes of AuNP: 24nm (a), 32 nm (b), and 36nm (c). Data were processed by means of the Software CAT.

References

[1] Cavalera S, Di Nardo F, Chiarello M, Serra T, Colitti B, Guiotto C, Fagioli F, Cagnazzo C, Denina M, Palazzo A, Artusio F, Pisano R, Rosati S, Baggiani C, Anfossi L. Bacterial ligands as flexible and sensitive detectors in rapid tests for antibodies to SARS-CoV-2. Anal Bioanal Chem. 2022 Feb 11:1–10. doi: 10.1007/s00216-022-03939-2. Epub ahead of print. PMID: 35149878; PMCID: PMC8853073.
